#  A Comparative Study of Intravenous Paracetamol and Fentanyl for Pain Management in ICU 

**Published:** 2013

**Authors:** Mehran Kouchek, Behnam Mansouri, Majid Mokhtari, Reza Goharani, Mir Mohammad Miri, Mohammad Sistanizad

**Affiliations:** a *Emam Hossein Hospital, Shahid Beheshti Medical University, Tehran, Iran.*; b *Department of Clinical Pharmacy, Scholl of Pharmacy, Shahid Beheshti Medical University, Tehran, Iran. *

**Keywords:** Pain, IV Paracetamol, IV fentanyl, Side effects

## Abstract

Pain in ICU patients should be managed effectively and safely. Fentanyl and Paracetamol are used frequently in ICU. However experience using IV Paracetamol in the setting of critically ill patients is limited. We evaluated the analgesic effect and adverse reactions of intravenous Paracetamol compared to Fentanyl in ICU patients with mild to moderate pain. Forty patients in a general ICU were randomized into two groups of IV Paracetamol and IV Fentanyl in a single blinded fashion. Pain was assessed by Visual Analogue Scale (VAS) before drug administration and six hourly for 48 h of 1 g IV Paracetamol every 6 h for 48 h in the first group and 25 μg Fentanyl intravenously every three hours for 48 h in the second group. Patients were monitored for significant adverse reactions particularly of CNS and hepatic nature. Results showed the age, sex and pain score before analgesia was matched in both groups. Pain scores were similar in both groups at 24 h 2.60 (± 1.2) and 2.40 (± 1.5) and at 48 h 2.25 (± 0.96) and 2.05 (± 1.1) in Paracetamol and Fentanyl groups respectively. Clinical and laboratory adverse reactions were also similar in both groups. The analgesic properties of Paracetamol and Fentanyl were similar in this study. We did not observe any significant adverse effects in the two groups. Clinical and laboratory findings including liver functions remained without any statistically significant difference in two groups. This study demonstrates intravenous Paracetamol may be as safe and effective as Fentanyl in ICU patients with mild to moderate pain.

## Introduction

Pain is one of the commonest symptoms of patients admitted to ICU. This should be managed safely and effectively not only from humane and patient comfort perspective but also for better overall care and healing of critically ill patients. 

There is heightened attention to pain management particularly after surgical interventions and procedures in recent times as we have better understanding of acute pain physiology, its complications and management modalities (1). 

Paracetamol is an effective and safe drug for managing mild to moderate pains (2). The oral and rectal form of this agent is widely used for pain and fever management in ICUs (3-6). The use of IV Paracetamol for pain management has gained some recognition in literature since its development with recent addition of this agent in formulary of many countries (7-13). 

NSAIDs are generally added to decrease the total doses of narcotics required for effective pain management. This is not without its harmfuleffects including GI bleeding as an added risk to ICU patients who are already prone to stress related gastric ulcer bleeding. 

Aghamir and coworkers compared IV Paracetamol with IV tramadol and demonstrated that Paracetamol is an effective and safe analgesic for acute postoperative pain management (14).

Peterson *et al *demonstrated that IV Paracetamol utilization can decrease the doses of narcotic analgesics in postoperative period of CABG patients (15).

Rod *et al. *undertook a study of the analgesic effects of morphine and IV Propacetamol for the management of postoperative pain compared in 239 pediatric patients. The analgesic effects were verified according to the physiological (Blood Pressure and Heart Rate) and behavioral response in this group of patients. This study indicated that IV Propacetamol could reduce doses of morphine amongst children during their postoperative period (16).

In another comparative study between intravenous Para cetamol and intramuscular meperidine in children after tonsillectomy, Alhashemi *et al*. showed that children treated withParacetamol were less drowsy and discharged earlier from the hospital as compared with meperidine group (17).

For the purpose of this study we used intravenous form of Paracetamol which was recently approved and registered by Pharmacopeia Committee of Ministry of Health in Iran.The comparative agent was Fentanyl which is the narcotic agent of first choice in our ICUs.

## Exprimental

*Patients and methods*

Forty postoperative ICU patients with mild to moderate pain entered this randomized prospective, single blinded study. After approval by the Institutional Review Board and obtaining informed written consent from patients or their surrogates they were randomized into two groups. First group received intravenous Paracetamo l1 g in 100 mL normal saline (Uni-Pharma pharmaceutical company, Greece) given in 15 min every 6 h and IV Fentanyl (Hameln Company Germany) 25 μg every three h, both for 48 h. Patients who had breakthrough pain or any need for supplemental analgesics were excluded from the study.

Patients had to have adequate mental status to be evaluated for severity of pain with mild to moderate pain based on a Visual Analogue Scale (VAS 2-5) (Diagram 1). Midazolam was the sedative agent which was used with equal doses as needed, for both groups. 

The exclusion criteria were history of allergy to Paracetamol or Fentanyl, contraindications to Paracetamol or opioids use, severe pain (VAS ≥ 5), GCS ≤13, renal disease (Serume Creatinine> 1.5 mg/dL), liver disease (liver enzymes >1.5 normal), chronic lung disease, hemorrhagic conditions, coagulation abnormalities, alcoholic patients, history of addiction to opioids, pregnancy and lactation. 

The analgesic effects of the drugs were measured on the basis of VAS and physiologic factors like PR (Pulse rate), RR (Respiratory rate), BP (Blood pressure) and perspiration assessed by clinical examination. Pain was assessed by ICU medical staff under supervision of ICU fellows and using VAS at the time zero *i.e*. before the analgesic administration and following study drugs administration every 6 h for 48 h. Other neurologic signs such as restlessness, hallucination, insomnia were recorded on hourly basis.

Blood pressure was measured and recorded 5 min before drug injections using non-invasive blood pressure monitors at 15, 30, 45, 60,120, 180 and 240 min after the injections then q4h for 48 h. Other Signs such as heart rate, respiratory rate, oxygen saturation and tympanic temperature were measured and absence or presence of perspiration was recorded.

All patients had to have normal liver, renal and coagulation profiles before entering the study. The laboratory investigations including CBC, BUN, Creatinine, Na, K, PT, PTT,ESR, INR, ALT, AST, FBS, BS, Bilirubin (total and direct) and Albumin were performed before and thereafter on 3^rd^ and 6^th^ day following drug administrations. 

Statistical analysis of the collected data was performed on SPSS (version 15) with the T-test method. Fisher exact test was used for comparative testing. The statistical significance with a p < 0.05 was considered valuable and all the results were recorded on the basis of percentage of the patient numbers or mean and standard deviation. 

## Results

A total of 40 patients were studied with 11 males and 9 females in Paracetamol group and 12 males and 8 females in IV Fentanyl group (p = 0.74). The age of patients were 42.4(± 16.2) and 41.7(± 15.4) in IV Paracetamol and IV Fentanyl respectively (p = 0.89).

The pain score based on VAS prior to the administration of analgesics (day 0) were 4.40 (± 0.8) and 4.35 (± 0.8) in Paracetamol and Fentanyl group respectively with a p-value of 0.84. 24 h after administration of study drugs the pain scores were 2.60 (± 1.2) and 2.40 (±.1.5) in Paracetamol and Fentanyl groups respectively (p = 0.64). At 48 h the pain scores were 2.25 (± 0.96) and 2.05 (± 1.1) in Paracetamol and Fentanyl groups respectively (p = 0.54). Improvement in the pain scores in each drug category at 48 h reached statistical significance with a p-value of < 0.0001 (Table and Figure 1).

**Table 1:** Visual Analogue Scale (VAS) for pain assessment in two groups of IV Paracetamol and IV Fentanyl during day 0, day 1 and day 2 of the study

**Variable VAS**	**Maximum**	**Minimum**	**IV Paracetamol ** **Mean (SD)**	**IV Fentanyl Mean (SD)**	**p-value**
pre-injection (Day 0)	6	3	4.40 (0.82)	4.35 (0.81)	0.84
24 h after Injection (Day 1)	5	1	2.60 (1.18)	2.40 (1.53)	0.64
48 h after injection (Day 2)	4	1	2.25 (0.96)	2.05 (1.09)	0.54
p-value Day 0 to 48 h			< 0.0001	< 0.0001	

VAS at 48 h in Paracetamol group was 3 in 7 (35%) patients, 2 in 8 (40%) patients and 5 (25%) patients were pain free. Whereas in Fentanyl group VAS was 4 in 3 (15%) patients, 3 in 3 (15%) patients and 8 (40%) patients were free (Figure 1).

The measured vital signs did not indicate any significant variations in heart rate, blood pressure, temperature or oxygenation, between the two groups during the course of study.

We did not notice any significant differences in the laboratory values between the two groups during the study period (Table 2).

**Table 2:** Important laboratory parameters observed in IV Paracetamol and IV Fentanyl groups on day 0, day 3 and day 6 of the study

**Lab Test** **(scale)**	**Drug for Infusion (IV)**	**Day 0** **(pre-infusion)** Mean (SD*)	**Day 3** **(3 day after infusion)** Mean (SD*)	**Day 6** **(6 day after infusion)** Mean (SD*)	**significance**
AST (mg/dL)	Paracetamol	42.9 (32.3)	42.3 (30.2)	41.2 (27.5)	NS
	Fentanyl	42.0 (41.5)	41.4 (35.9)	43.5 (36.8)	
	p= value	0.94	0.93	0.82	
ALT (mg/dL)	Paracetamol	30.9 (27.7)	40.2 (29.4)	41.5 (29.9)	NS
	Fentanyl	36.4 (37.9)	39.7 (38.0)	41.5 (38.1)	
	p = value	0.60	0.96	1.00	
Bilirubin Total (mg/dL)	Paracetamol	1.01 (0.89)	0.87 (0.48)	0.78 (0.3)	NS
	Fentanyl	0.98 (0.78)	0.87 (0.51)	0.74 (0.35)	
	p= value	0.91	1.00	0.70	
Bilirubin Direct (mg/dL)	Paracetamol	0.31 (0.27)	0.35 (0.28)	0.28 (0.16)	NS
	Fentanyl	0.31 (0.28)	0.35 (0.27)	0.38 (0.28)	
	p= value	0.70	0.96	0.20	
PT (Second)	Paracetamol	14.0 (1.8)	14.6 (3.3)	15.6 (4.3)	NS
	Fentanyl	13.4 (1.02)	13.4 (1.2)	13.5 (1.04)	
	p= value	0.18	0.14	0.044	
PT (Second)	Paracetamol	36.9 (11.0)	34.9 (7.9)	34.7 (6.1)	NS
	Fentanyl	34.7 (3.8)	35.5 (3.8)	34.5 (4.7)	
	p= value	0.39	0.73	0.92	
INR (Number)	Paracetamol	1.17 (0.28)	1.3 (0.45)	1.2 (0.39)	NS
	Fentanyl	1.17 (0.15)	1.16 (0.15)	1.15 (0.16)	
	p= value	0.97	42.3 (30.2)	0.14	

Adverse drug reactions such as fever, hypotension, gastrointestinal bleeding, headaches, agitation, hallucinations, skin reactions, liver and renal complications were monitored closely. One patient in Paracetamol group developed transient skin rash which resolved without treatment. We did not observe any significant neurological adverse reaction in either group. 

## Discussion

Paracetamol is a safe and effective analgesic agent for mild to moderate pain. The oral and rectal forms of this drug are commonly used for pain management. The intravenous form of this agent passes easily through the blood brain barrier and shows its central analgesic effects within 15-20 min which starts to decline after 4 h of administration.

Although this agent represents a relatively good safety profile, there is scarcity of studie sregarding its IV use in critically ill patients of ICUs.

We undertook this prospective randomized study to examine the efficacy and side effect profile of IV Paracetamol compared to IV Fentanyl in postoperative patients with mild to moderate pain in ICU.

Our study did not indicate any statistically significant difference between the IV Paracetamol and IV Fentanyl groups in pain scores at 24 or 48 h. Neither did we note any significant difference in physiological parameters nor side effect profiles both clinical and laboratory, between the two groups.

Of note there was no neurological or liver function abnormalities of any significance in the Paracetamol group with doses used in our study.

In a study comparing one g IV Paracetamol and 2 g IV Propacetamol which showed comparable efficacy, less pain and discomfort was observed over the injection site with Paracetamol (6). We also did not observe any pain over Paracetamolinjection site in our study.

In another study, it was demonstrated that administration of 1 g Paracetamol intravenously before hysterectomy resulted in better postoperative pain control and lead to decreased use of morphine (18).

A study in patients undergoing lower segment cesarean section, in which IV Paracetamolwas compared with oral Ibuprofen, as the analgesic supplementation agent to morphine, indicated that patients in IV Paracetamol had better pain control compared to Ibuprofen group (19).

According to another work conducted by Cakan *et al*. on 40 patients in a randomized double blind clinical trial, studying post lumbar laminectomypain management, in addition to morphine administration, one group received one g IV Paracetamol every six hours and the other group received IV placebo. The amount of IV morphine used did not show any significant difference in the two groups. Vomiting was observed less frequently in the placebo group. The Paracetamol group had better pain management profiles than the placebo group (20). In another work carried out by Sabetkasaei *et al*. the role of alternative agents to opioids-based drugs in acute pain management was highlighted again (21). 

Our study, utilizing VAS, indicated similar pain scores between the two groups of intravenous Paracetamol and Fentanyl on days 1 and 2. However at 48 h, more patients were pain-free (VAS 0-1) in Fentanyl group which did not reach statistical significance. We did not observe any adverse effects such as vomiting in our studied patients. 

Limitations of our study such as small sample size, lack of case diversity, lack of placebo arm, single blinded nature, short follow up duration and absence of baseline analgesic agent to compare IV Fentanyl and IV Paracetamol over its use, were reasons we could not draw further conclusions as to the other characteristics Paracetamol use in ICU. 

However this small study indicates similar pain management properties of IV Paracetamol and IV Fentanyl in mild to moderate pain management in ICU.

## Conclusion

Intravenous Paracetamol appeared as effective and safe as Fentanyl in the management of mild to moderate pain in ICU patients. In doses used in our study, we did not observe any significant intolerance or hepatic dysfunction in the Paracetamol group.

IV Paracetamol could be considered another analgesic agent in ICU pain management armamentarium.

*Financial disclosure*

Paracetamol injections (APOTEL)® were provided by the manufacturer Uni-Pharma pharmaceutical company, Greece. This research has been supported by Behavior Sciences Research Center of Shahid Beheshti Medical University, Tehran/Iran.
